# Screening and Application of DNA Aptamers for Heparin-Binding Protein

**DOI:** 10.3390/molecules29081717

**Published:** 2024-04-10

**Authors:** Xi Zhou, Yingying Cao, Xiaocui Huang, Shuqian Qiu, Xinran Xiang, Huimin Niu, Li Chen, Shuiliang Wang, Zhenyu Lin, Shenghang Zhang

**Affiliations:** 1 Fujian Key Laboratory of Aptamers Technology, Fuzhou General Teaching Hospital (the 900th Hospital), Fujian University of Traditional Chinese Medicine, Fuzhou 350025, China; xizhou939@163.com (X.Z.); caoyy0906@163.com (Y.C.); qiusq1221@163.com (S.Q.); xiangxr2022@163.com (X.X.); newgirl4440@163.com (H.N.); chenlii0104@126.com (L.C.); shuiliang.wang@xmu.edu.cn (S.W.); 2 Department of Clinical Laboratory Medicine, Fuzhou General Clinical Medical School, Fujian Medical University, Fuzhou 350025, China; 3 Department of Science Research and Training, Fujian Institute of Education, Fuzhou 350001, China; xchuang147@163.com; 4 MOE Key Laboratory of Analysis and Detection for Food Safety and Biology, Fujian Provincial Key Laboratory of Analysis and Detection Technology for Food Safety, College of Chemistry, Fuzhou University, Fuzhou 350116, China

**Keywords:** sepsis, aptamers, biosensors, heparin-binding protein, rolling circle amplification

## Abstract

Rapid detection of heparin-binding protein (HBP) is essential for timely intervention in sepsis cases. Current detection techniques are usually antibody-based immunological methods, which have certain problems, such as complexity and slow detection, and fall short in meeting the urgency of clinical needs. The application of an aptamer can address these concerns well. In this study, HBP-specific DNA aptamers were screened first. Among which, Apt-01, Apt−02, and Apt−13 had a high affinity for HBP, exhibiting impressive K_D_ values of 3.42, 1.44, and 1.04 nmol/L, respectively. Then, the aptamer of HBP and its partially complementary primer probe were combined to form double-stranded DNA (dsDNA) and synthesize a circular DNA template. The template is complementary to the primer probe, but due to the presence of dsDNA, ExoIII cleaves C2-13 as an RCA primer probe, rendering the template unable to recognize the primer probe and preventing the RCA reaction from proceeding. When the target is present, it competes with the adapter for recognition and releases C2-13, exposing its 3′ end. After initiating the RCA at room temperature and reacting with SYBR GreenII at 37 °C for 20 min, fluorescence changes can be observed and quantitatively analyzed at a 530 nm wavelength, achieving quantitative biological analysis. Apt-01 was used to develop a fluorescent biosensor for HBP detection, which exhibited a good linear range (0.01 nmol/L to 10 nmol/L) and detection limit (0.0056 nmol/L). This advancement holds the potential to lay a solid groundwork for pioneering sensitive and specific methods for HBP detection and to significantly enhance the diagnostic processes for sepsis.

## 1. Introduction

Sepsis, a clinical disorder characterized by significant physiological, pathological, and biochemical imbalances secondary to infection, has a substantial global health burden, with high morbidity and mortality rates [1]. In Chinese intensive care units (ICUs), sepsis impacts nearly 20% of patients and is associated with a daunting 90-day mortality rate of 35.5% [2]. As there is currently no specific diagnostic tool for sepsis, the progression and prognosis of sepsis is monitored in the clinical setting mainly by virtue of the Sequential Organ Failure Assessment (SOFA) score [3,4], whereas the laboratory diagnosis of sepsis is mainly based on routine clinical markers such as C-reactive protein and procalcitonin, which while valuable, are insufficiently specific for the accurate discernment of sepsis, and this highlights the necessity for more definitive biomarkers [5,6,7]. Heparin-binding protein (HBP), a mediator secreted by activated neutrophils, is a biological indicator in the progression of the cascade. Neutrophils release chemotactic substances upon activation by infectious agents, causing the secretion of HBP, promoting cytoskeletal changes. These changes increase vascular permeability, facilitating neutrophil extravasation to the infectious focus [8]. HBP is discharged from azurophilic granules, exacerbating tissue cell migration and inflammatory mediator synthesis [9,10]. In the serum of healthy individuals, the concentration of HBP is below 10 ng/mL (0.26 nmol/L). Some studies have shown that the concentration of HBP in the serum of patients with infectious diseases will increase (more than 10 ng/mL), and especially in patients with sepsis, it is even higher than 100 ng/mL (2.6 nmol/L) [11,12]. Therefore, HBP’s role is increasingly recognized within the pathogenesis of infectious diseases, specifically sepsis [13].

Current HBP diagnostic modalities primarily rely on antibody-based immunoassays, including biotin–avidin system-enhanced time-resolved fluorescence immunoassay (BA-TRFIA) [14], latex immunoturbidimetry [15], enzyme-linked immunosorbent assay (ELISA) [16], and the emergent dry immunofluorescence assay [17]. These approaches, while effective, are hindered by the high costs of antibodies and the need for cryogenic storage, which limit their utility in point-of-care (POC) settings. Therefore, there is an urgent need for rapid, cost-effective, and sensitive HBP detection strategies for early sepsis detection in a clinical setting.

**Figure 1:**
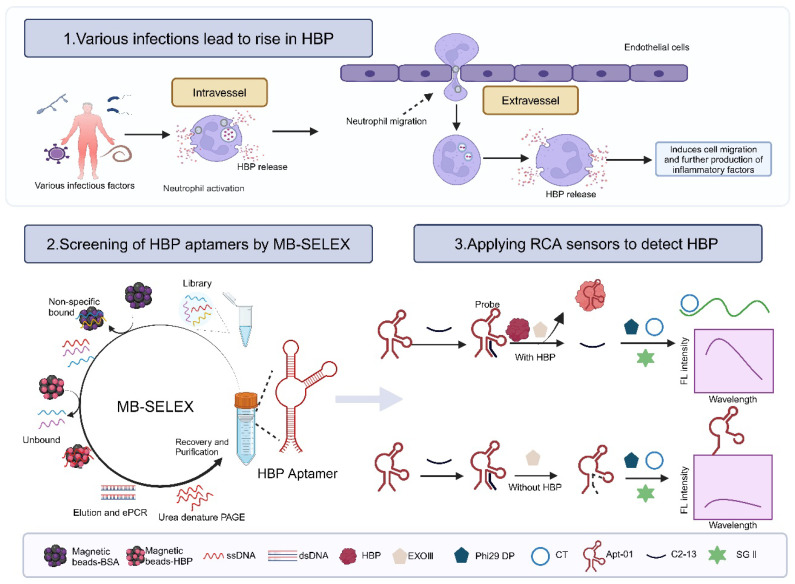
Principle of constructing a fluorescent biosensor based on aptamer and RCA technology for detecting the biomarker HBP in sepsis. Created with BioRender.com.

Nucleic acid aptamers, often known as “synthetic antibodies”, owing to the aptamers’ potential to assume various folded conformations, their recognition of targets being similar to the antigen–antibody interaction, and their specificity and affinity being comparable to traditional antibodies, offer a promising alternative to antibodies [18,19,20]. Systematic Evolution of Ligands by Exponential Enrichment (SELEX), the cornerstone for aptamer selection, encompasses an array of methodologies, including magnetic bead-SELEX (MB-SELEX) [21], graphene oxide-SELEX (GO-SELEX) [22], capillary electrophoresis-SELEX (CE-SELEX) [23,24], CELL-SELEX [25], Tissue-SELEX [26], Post-SELEX [27], and Gold-SELEX [28]. Among these, magnetic beads have achieved prominence as solid-phase supports in MB-SELEX for immobilizing targets because of their expansive surface area, robust stability, ease of surface modification, minimal sample requirements, and straightforward operational procedures [29,30]. In this study, MB-SELEX technology was applied to isolate three HBP-specific nucleic acid aptamers and the selected aptamer was used to construct a fluorescent biosensor by combining rolling circle amplification (RCA) signal amplification technology to demonstrate the potential application of the aptamer (Figure 1).

## 2. Results and Discussion

### 2.1. Selection of Aptamers

**Figure 2:**
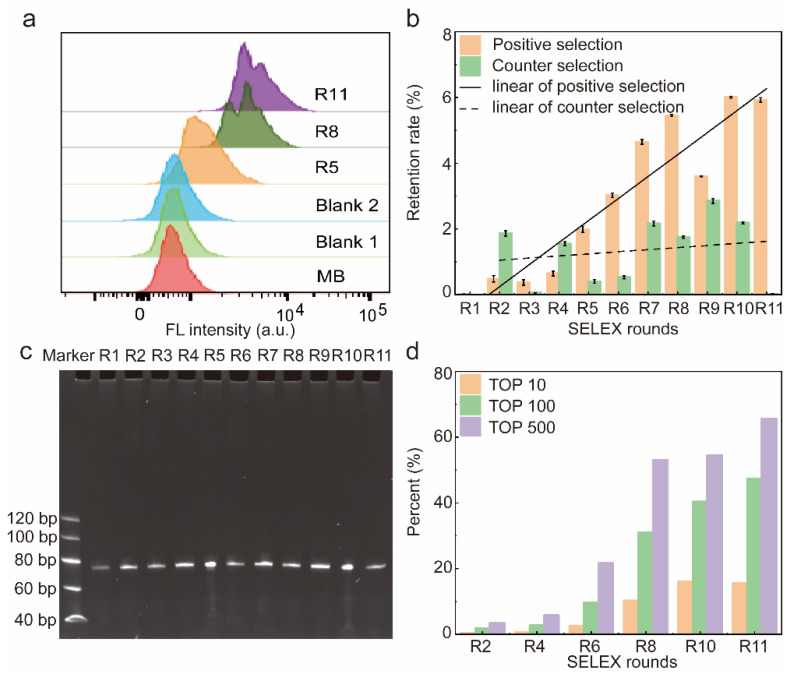
Characterization of the resulting library. (**a**) Affinity of libraries characterized by flow cytometry. (**b**) Recovery rate of the 11 rounds of screening. Error bars are standard error of the mean, n = 3 for each condition. (**c**) Eleven rounds of libraries were confirmed to be free from contamination via 8% polystyrene amide gel electrophoresis. (**d**) The abundance of top 10, 100, and 500 sequences in the whole library of different SELEX rounds. A total of 600,000 sequences were analyzed in the high-throughput sequencing.

A total of 11 rounds of screening were performed in this work, and the affinity of the libraries for HBP was assessed by flow cytometry. The fluorescence shift of the library was more pronounced as the number of screening rounds increased, indicating that the amount of single-stranded DNA (ssDNA) bound to HBP-encapsulated MB increased with the number of selection rounds (Figure 2a). The fluorescence intensity shown by R5 displayed a significant rightward shift with respect to MB-HBP (Blank2), indicating that R5 showed a significant increase in its affinity for HBP that leveled off from round 8. In contrast, the fluorescence intensity of MB-R11 (Blank1) remained unchanged after treatment with the most fluorescently shifted R11 compared to the MB control, indicating that the fluorescence shift was due to the binding of the library to the HBP. To quantitatively assess the retention rate of the library at each screening stage, this study generated varying concentrations of the initial Lib76 library to obtain a standard curve. The derived regression equation, y = −0.21068x + 5.03509 with an R2 value of 0.9993, correlated the logarithmic value of the initial concentration (y) with the quantification cycle (Cq) value (x) (Figure S1). Figure 2b illustrates the retention rate of the library over each selection round. At R1–R8, the retention of ssDNA libraries for positive selection gradually increases with the number of screening rounds and reaches a plateau at R8. After eight rounds of screening, this work used a reduction in the positive selection incubation time and an increase in the number of washes to enhance the screening efficiency. The positive selection incubation time was reduced from 60 min to 45 min, and the number of washing steps was increased from four to six during the ninth round. This adjustment resulted in a short-term decrease in the positive selection recovery rate of the ninth round, which normalized in the subsequent tenth round. On the contrary, R1 to R11 did not show a significant upward trend in the retention of counter selection, and the binding rate of R11 to the counter selection reached a minimum, indicating that the candidate has a high affinity and specificity for HBP.

Therefore, after 11 rounds of screening, the ssDNA library was successfully enriched, and in order to save the cost of the experiment, R2, R4, R6, R8, R10, and R11 were selected to be sequenced and analyzed in the next step.

The electrophoretic analysis confirmed the consistent size of the library fragments from rounds 1 to 11, with each round yielding a library of 76 nucleotides (nt) (Figure 2c). After eleven rounds of meticulous screening, three high-affinity aptamers were successfully obtained.

### 2.2. High-Throughput Sequencing

After R11, the SELEX process was completed and R2, R4, R6, R8, R10, and R11 were selected for high-throughput sequencing. After discarding sequences with mismatches in constant regions and sequences with random regions of lengths different from 36 nt, the frequency distribution of the random N36 portion of the 6 × 105 sequences was analyzed. In total, 312 sequences were represented more than 500 times in R11, 182 sequences were displayed more than 1000 times, 34 sequences were expressed more than 5000 times, and 14 sequences were expressed more than 10,000 times (Figure 2d). In addition, the abundance of the top 10, 100, and 500 sequences increased with the number of selection rounds. After 11 rounds of selection, the top 10 sequences were enriched to 15.47% of the 605,400 sequences, indicating that the complexity of the circular library decreased as SELEX progressed. Supplementary Table S4 outlines the 100 most prevalent sequences identified via high-throughput sequencing and their corresponding abundances.

### 2.3. Affinity and Specificity of Selected Aptamers

In this analysis, in order to enable a more straightforward comparison of the sequence homology, the terminal primer sequences were removed, centering the analysis on the core random sequences, followed by the homology comparison of the first 30 sequences using ClustalX 2.1 and MEGA 7 software, which separated the sequences into seven distinct families according to their similarity, with each family exhibiting a number of similar sequences (Figure S2). Figure S3 sheds light on the conserved and variable regions within the 30 sequences analyzed. Specifically, the conserved nucleotide sites (TTCACCTACCG) were situated at the 5′ terminus of the random sequences, albeit with specific mutations. Then, a collection of 14 candidate aptamers (Apt-01, 02, 03, 04, 05, 07, 08, 09, 12, 13, 14, 15, 18, 20) was generated by selecting the two most dominant sequences from each family of sequences and the M-fold web server was used to perform Gibbs free energy calculations and predictions of the secondary structure, which inform their thermodynamic stability. The average Gibbs free energy value of the 14 candidate aptamers in Table S5 is −11.62 ± 1.53, indicating that these 14 candidate aptamers have robust stability. In addition, surface plasmon resonance (SPR) was utilized to detect the stability and affinity of the 14 candidate aptamers for HBP, as indicated by the response value (RU) (Table S5). Notably, seven of them showed response values (RUs) of more than 100 RUs to the HBP protein as measured by SPR. Based on these response values and Gibbs free energy calculations, the aptamers with RUs exceeding 150 RUs were further screened, leading to the selection of Apt-01, Apt−02, and Apt−13 for analysis. The secondary structures of the three aptamers are shown in Figure S4.

**Figure 3:**
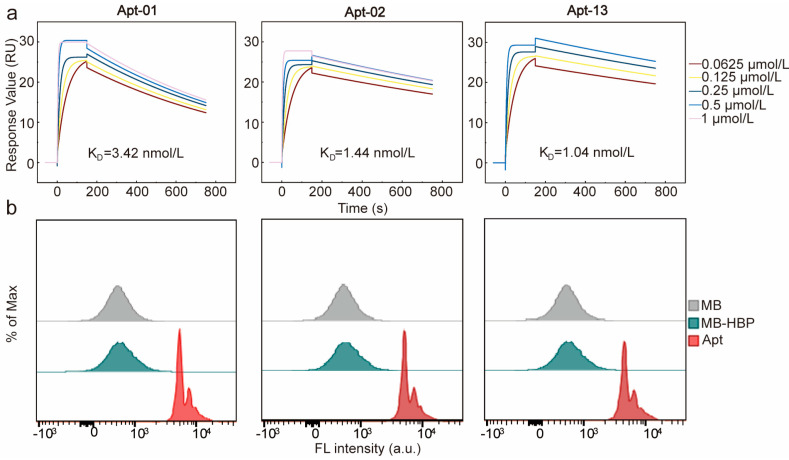
Characterization of the affinity and specificity of Apt-01, Apt−02, and Apt−13. (**a**) Characterization of K_D_ values through surface plasmon resonance. (**b**) Affinity verification of the three aptamers using flow cytometry.

Furthermore, the equilibrium dissociation constants (K_D_) of the three aptamers were determined using SPR (Figure 3a). The K_D_ values of the three aptamers were 3.42, 1.44, and 1.04 nmol/L, respectively, which were all at the nanomolar level, suggesting that all of the three aptamers had a good affinity to HBP. The affinity and specificity of the candidate aptamers to HBP were also confirmed by flow cytometry analysis. A pronounced rightward shift in fluorescence intensity was identified when MB-HBP was treated with the fluorescein amidite (FAM)-labeled aptamers, substantiating their potent affinity for HBP (Figure 3b). The circular dichroism (CD) spectrum of the three aptamers was detected before and after the interaction with HBP in PBS buffer. The results revealed that the three aptamers characterized constitute a family of B-DNA forms with common global features, which exhibit a positive long wavelength band or bands centered around 260–280 nm, accompanied by a corresponding negative band at approximately 240–245 nm [31]. After the reaction of the aptamer and HBP for 30 min, the spectrum of the aptamer shifted significantly at 280 nm, indicating that there was a direct physical interaction between the aptamer and HBP (Figure S5).

### 2.4. Principle and Feasibility of Fluorescence Biosensor for HBP Based on the Selected Aptamer

**Figure 4:**
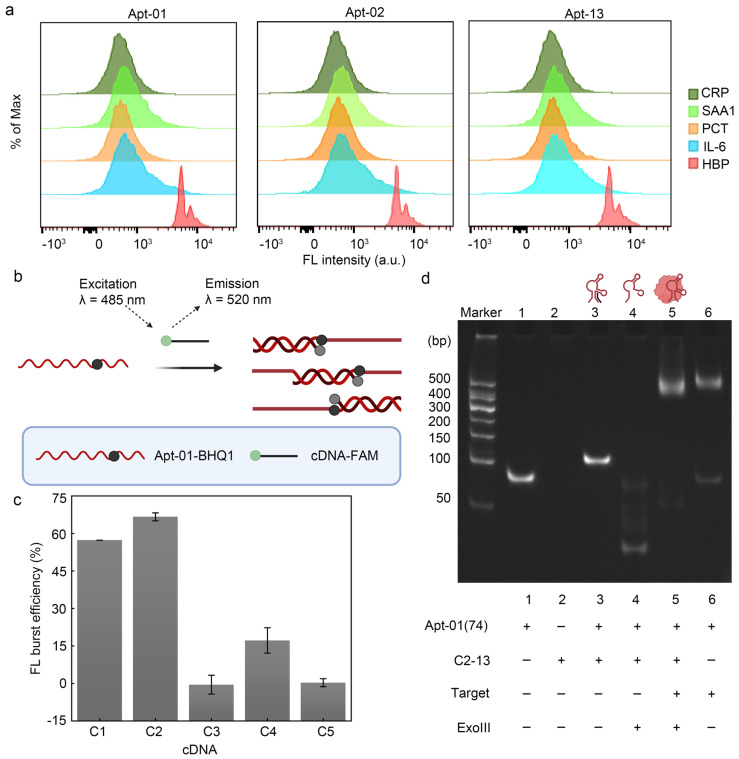
Feasibility of fluorescence biosensor. (**a**) Verification of the three aptamers using flow cytometry. (**b**) FRET validates the interaction of Apt-01 with cDNA. Created with BioRender.com. (**c**) C1 and C2 can hybridize stably with the aptamer. Error bars are standard error of the mean, n = 3 for each condition. (**d**) Characterization of polypropylene gel electrophoresis.

To build a fluorescence biosensor for HBP based on the selected aptamer, the specificity of the selected aptamer was explored. When the aptamer interacted with the MB modified by the remaining four alternative proteins (CRP, PCT, SAA1, IL-6) (Figure 4a), the fluorescence shift of its samples was negligible, suggesting that the three aptamers screened could not have a significant bind to these four alternative proteins, confirming the specificity of the aptamer–HBP interaction.

Given that the K_D_ values and flow cytometry profiles of the three aptamers were remarkably similar, Apt-01 was selected for further functional validation by comparing the enrichment levels of the aptamers and SPR response values (Table S5). For the RCA reaction to proceed smoothly, fluorescence resonance energy transfer (FRET) and polyacrylamide gel electrophoresis (PAGE) were firstly used to verify whether Apt-01 formed a stable double-stranded structure with partially complementary DNA (cDNA) and whether this double-stranded structure would lead to the freeing of the cDNA due to the incorporation of HBP. FAM acted as an energy donor, while black hole quencher 1 (BHQ1) acted as the corresponding bursting agent. The cDNA was modified with FAM, while Apt-01 was labeled with BHQ1. The proximity of FAM and BHQ1, due to the hybridization of the aptamer with the cDNA, enabled a FRET effect, effectively silencing the FAM fluorescence signal (Figure 4b). The significant fluorescence bursts for C1 and C2 upon interaction with Apt-01 demonstrated the successful hybridization and formation of double-stranded DNA (dsDNA) probes, especially in the case of C2 (Figure 4c). Conversely, C3, C4, and C5 displayed suboptimal fluorescence quenching, suggesting their inability to consistently disrupt the aptamer structure for stable double-strand formation. Upon selection of the C2 construct for its competitive fluorescence sensing capabilities, the length of C2 was adjusted to enhance its affinity to HBP and to improve the stability of the probe. The length-adjusted C2 was named C2-13, containing 16 oligodeoxyribonucleotides.

In order to enable the freeing of C2-13 from dsDNA in the presence of HBP for the subsequent RCA reaction, this work therefore removed the two adenines (A) at the 3′ end of Apt-01 (Apt-01 used for the subsequent sensor build was 74 nt, named Apt-01(74), described in Table S1), which not only facilitated the digestion of the excess dsDNA by exonuclease III (Exo III), but also prevented the addition of the circular template (CT) from competing for C2-13 in the dsDNA when HBP was not present and leading to the onset of the RCA reaction, thus reducing a certain amount of background signal. This work investigated the optimal concentration ratio of C2-13 to Apt-01(74). The addition of the Apt-01(74) to C2-13 resulted in fluorescence quenching, with the quenching effect reaching a near-equilibrium state at a 1:1 concentration ratio. Further elevation of the concentration ratio to 1:2 did not yield a significant enhancement in quenching (Figure S6). Therefore, considering the magnitude of background fluorescence intensity and cost-efficiency, the 1:1 concentration ratio of cDNA to aptamer was deemed as the most favorable condition.

### 2.5. Characterization of Polyacrylamide Gel Electrophoresis

In order to verify that HBP was able to compete for C2-13 from the Apt-01(74)/C2-13 complex strand, polyacrylamide gel electrophoresis was used in this work. Higher bands were observed in lane3 compared to lane1, indicating that the Apt-01(74)/C2-13 complex strand hybridized efficiently and higher-molecular-weight dsDNA was obtained. Bright bands were observed higher up in lane5 in the presence of HBP compared to the absence of added HBP (lane4), indicating that the combination of Apt-01(74) with HBP could save Apt-01(74) and C2-13 from Exo III digestion (Figure 4d). In addition, the successful preparation of CT was also demonstrated by polyacrylamide gel electrophoresis experiments (Figure S7).

### 2.6. Optimization of the Reaction Conditions

**Figure 5:**
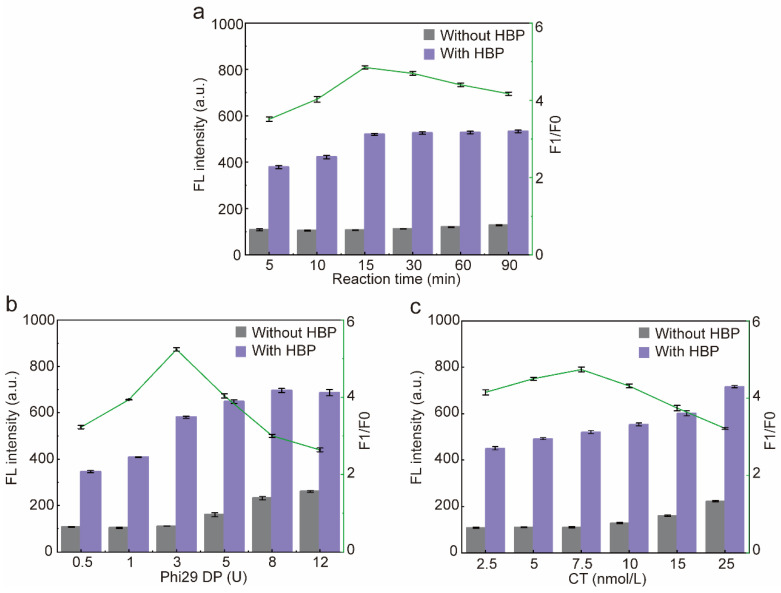
Optimization of the sensor. (**a**) Optimization of the RCA reaction time. (**b**) Optimization of the activity unit of Phi29 DP. (**c**) Optimization of the concentration of CT input into the sensor. F1/F0: F1 means the fluorescence intensity of the experimental group (with HBP), F0 means the fluorescence intensity of the blank group (without HBP). Error bars are standard error of the mean, n = 3 for each condition.

In order to obtain the best sensor performance, the RCA reaction time and the concentration of Phi29 DP and CT were optimized. Firstly, the RCA reaction time was optimized, and the fluorescence intensity of the blank group did not change significantly with the increase in the reaction time, while the fluorescence intensity of the experimental group was gradually enhanced and reached the plateau at 15 min, so the reaction time of RCA was determined to be 15 min (Figure 5a). The fluorescence intensity of the blank group and the experimental group gradually increased with the increase in the dosage of Phi29DP, and the fluorescence intensity of the experimental group reached a plateau at 8 U. However, when the dosage of Phi29 DP was 3 U, F1/F0 was the highest, and a further increase in the dosage of Phi29 DP would result in a greater increase in the fluorescence intensity of the blank group than that of the experimental group due to the nonspecific amplification of the reaction system, thus resulting in a decrease in F1/F0. Therefore, Phi29 DP was determined to be 3U (Figure 5b). Subsequently, the CT concentration was optimized, due to the fact that CT belongs to ssDNA, so it could be stained by SYBR Green II; therefore, the fluorescence intensity of the blank group and the experimental group increased gradually with the increase in the concentration of CT. When the concentration of CT was 7.5 nmol/L, F1/F0 was the highest; afterwards, when the CT exceeded 7.5 nmol/L, due to the nonspecific amplification of the reaction system, it resulted in an increase in the fluorescence intensity in the blank group greater than that of the experimental group, which led to a decrease in F1/F0. Therefore, the optimum CT concentration was determined to be 7.5 nmol/L (Figure 5c).

### 2.7. Performance of the Selected Aptamer for HBP Determination

**Figure 6:**
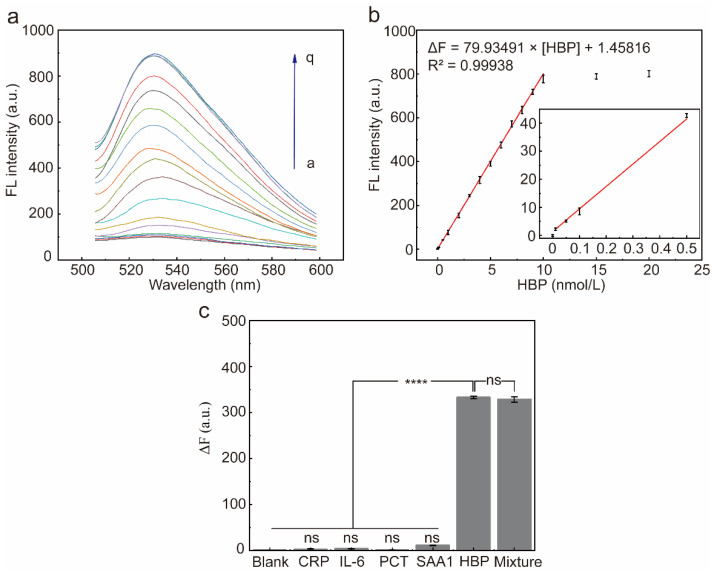
Characterization of sensor performance. (**a**) Fluorescence spectra of the RCA reaction system after incubation with different concentrations of the HBP (a~q: 0, 0.01, 0.05, 0.1, 0.5, 1, 2, 3, 4, 5, 6, 7, 8, 9, 10, 15, 20 nmol/L, respectively) for 15 min. (**b**) ΔF at 530 nm and HBP target concentrations from 0 to 20 nmol/L. Red line: linear correlation between fluorescence intensities and HBP concentrations in the range of 0.01–10 nmol/L. Error bars are standard error of the mean, n = 3 for each condition. (**c**) ΔF at 530 nm after addition of each of the five proteins to the reaction system. ΔF = F1 − F0, F1 is the fluorescence intensity in the presence of HBP, and F0 is the fluorescence intensity in the absence of HBP. Error bars are standard error of the mean, n = 3 for each condition. One-way ANOVA for multiple comparisons was used to compare each group to the condition with HBP and without HBP. ns, *p* > 0.05; ****, *p* < 0.0001.

The fluorescence response of the system was recorded after the addition of different concentrations of HBP under optimal reaction conditions (Figure 6a). The ΔF at 530 nm incrementally increased in tandem with the increasing concentrations of HBP and reached a plateau at 10 nmol/L. There was a robust linear relationship between the ΔF and the HBP concentration (the range of 0.01 nmol/L to 10 nmol/L), the fitted regression equation was ΔF = 79.93491 × [HBP] + 1.45816, and the calculated detection limit was 0.0056 nmol/L (3-fold deviation according to the blank response rule), where [HBP] denotes the concentration of HBP (Figure 6b).

To assess the selectivity of the HBP-initiated RCA response, four non-targeted inflammatory proteins were used to initiate the RCA response, including CRP, PCT, IL-6, and SAA1. There was no significant difference in ΔF produced by the four non-targeted inflammatory proteins when compared to the blank group (Figure 6c, *p* > 0.05), indicating that these inflammatory proteins did not interfere with the detection of HBP. In contrast, when the HBP-containing samples were added to the reaction system, the fluorescence intensity at 530 nm increased significantly, and the ΔF of the HBP-containing experimental group was significantly different compared with the blank group and the remaining four non-target inflammatory proteins group (Figure 6c, *p* < 0.0001). In conclusion, the results showed that the sensor has good selectivity against HBP and can distinguish HBP from other inflammatory proteins.

### 2.8. Accuracy of the Proposed Fluorescent Sensor in Real Sample Detection

As shown in Table 1, compared with healthy individuals undergoing physical examinations, the serum HBP concentration in sepsis patients significantly increased, which is consistent with the clinical diagnostic results. This indicates that the fluorescence sensor constructed in this work has good accuracy, can measure the concentration of HBP, and can be used for the diagnosis of sepsis.

## 3. Discussion

Sepsis is a systemic inflammatory response syndrome caused by infection, commonly seen in patients with severe trauma or infectious diseases [32]. According to statistical data, sepsis exhibits a high incidence and mortality rate, rendering it the primary cause of fatality among critically ill patients in intensive care units [33,34]. Furthermore, survivors often experience a significant impact on their quality of life, frequently accompanied by neuromuscular weakness, functional decline, depression, anxiety, and post-traumatic stress syndrome [35]. Timely and accurate diagnosis of sepsis is therefore crucial for reversing its adverse clinical course.

In clinical studies, HBP has also been confirmed as a biomarker for various bacterial infections. Elevated levels of HBP in cerebrospinal fluid and urine are associated with bacterial meningitis and urinary tract infections, respectively [36]. Previous studies have demonstrated that the plasma concentrations of HBP can exhibit significant elevation within 1–2 h during acute bacterial infections, while viral infections generally result in either no increase or only a slight one [37]. Several guidelines have elucidated the role of HBP in the early diagnosis and prognosis of sepsis [38]. Therefore, HBP is primarily utilized as a potential biomarker for sepsis [39,40]. Compared to PCT and CRP, HBP exhibits earlier and more sensitive elevation, making it imperative to develop faster and more portable detection methods for this marker [41].

Currently, diagnostic methods for heparin-binding protein (HBP) primarily rely on antibody-based immunoassays, such as the double-antibody sandwich method based on immunofluorescence technology with a detection range of 5.9–300 ng/mL and the traditional enzyme-linked immunosorbent assay (ELISA) with a detection range of 0.156–10 ng/mL and detection limit of 0.054 ng/mL. These methods are hindered by their high cost and need for low-temperature storage, limiting their application in point-of-care testing (POCT) environments. Therefore, there is an urgent need for innovative HBP detection strategies that are not only simple and cost-effective but also possess the sensitivity required for early sepsis detection in clinical settings. Therefore, we built a clinical diagnostic method for HBP with an aptamer. Firstly, we screened the aptamer of HBP. We obtained three high-affinity and strong specificity aptamers of HBP through MB-SELEX technology. This is the first report about screening an HBP aptamer. Secondly, we used the aptamers to construct clinical diagnostic methods. Rolling circle amplification takes a small circular oligonucleotide as a template and dNTPs as raw materials to amplify a long repeated single-stranded DNA/RNA under the action of DNA/RNA polymerase. The RCA method has been widely used in genomics, proteomics, molecular diagnosis, biosensing, drugs, and other fields. For example, RCA combined with surface-enhanced Raman scattering technology is used to detect Vibrio parahaemolyticus in water samples. RCA primers are brought into the reaction system for RCA reaction by double-antibody sandwiching, resulting in a long ssDNA with repeated sequences to capture Au@Ag probes, which improves the enrichment of nanoparticles in the reaction system and enhances the Raman signal. The detection limit of this method reaches 1 CFU/mL [42]. Xu designed the complementary sequence of palindromic DNA on the RCA template and induced the RCA reaction by Let-7a to produce a large number of ssDNA containing palindromic sequences. The palindromic sequences folded themselves into hairpin DNA containing a double-stranded structure, and SYBR Green I was embedded in the double-stranded DNA to produce a strong fluorescence signal for detection. The results showed that the detection limit of this method was 6.4 pmol/L under the optimal conditions [43]. Huang established a new type of electrochemical sensor based on RCA signal amplification for the rapid and sensitive detection of ochratoxin A (OTA). By introducing the RCA amplification system, more molecular beacons were fixed on the gold electrode plate for electrochemical detection. Under the optimal conditions, the detection limit of OTA was 0.065 pg/mL, and this method could be successfully applied in the detection of wine samples [44]. Huang established a cyclic chain displacement reaction combined with an RCA electrochemical sensor for detecting hepatitis B virus. Specific hepatitis B virus DNA was used as the “bridge” between the gold electrode plate and the RCA initiation chain, and the initiation chain was extended under the action of DNA polymerase to replace hepatitis B virus DNA and serve as the next “bridge”. The results showed that the detection limit of this method for hepatitis B virus was 2.6 amol/L, and the linear range was 10–700 amol/L [45].

RCA technology can be flexibly combined with materials, dyes, etc. In our experiment, we proposed a fluorescence sensing strategy based on an aptamer, using isothermal rolling circle amplification signal amplification caused by target competition. The detection range of this method is 0.01~10 nmol/L, and the detection limit is 0.0056 nmol/L. The RCA-FL method is simple, and its high sensitivity and specificity make it helpful for the early screening, severity, and prognosis assessment of sepsis patients. It is expected to become a new clinical detection platform and be applied in primary clinical institutions to achieve the accurate quantification of HBP, which is crucial for reducing the incidence of multiple organ failure and mortality. However, the RCA strategy method still needs to be improved, such as the high requirements of DNA ligase on the reaction system, the cumbersome operation, the need for phosphorylated DNA in the reaction to build a loop, and the long reaction time.

Therefore, based on the self-selected HBP aptamer, a fluorescence sensing strategy using the target competition-induced RCA-FL method is proposed. RCA-FL can be used to monitor patients at increased risk of bacterial infection, which can lead to the timely diagnosis of bacterial infection, early detection of sepsis, and timely antibiotic treatment.

## 4. Experimental Section

### 4.1. Materials and Reagents

Heparin-binding protein (HBP), Procalcitonin (PCT), C-reactive protein (CRP), Serum amyloid A1 (SAA1), Interleukin-6 (IL-6), and Bovine serum albumin (BSA) were all obtained from Novoprotein Co., Ltd. (Suzhou, China). A protein screening kit V1.0 was acquired from Anhui Aptamy Biotechnology Co., Ltd. (Hefei, AnHui, China). Phi29 DNA polymerase (Phi29 DP), 10× Phi29 DP buffer, deoxyadenosine triphosphates (dNTPs), exonuclease I (Exo I), exonuclease III (Exo III), Dulbecco’s phosphate-buffered saline buffer (DPBS), phosphate-buffered saline buffer (PBS), butanol, ultrapure water, and all other chemicals were analytical grade and acquired from Sinopharm Chemical Reagent Co., Ltd. (Shanghai, China).

### 4.2. Random Library and Primers

The random library, which encompassed the protein screening kit V1.0, was termed libP1-76nt. This library was composed of a 76-nucleotide (nt) single-stranded DNA (ssDNA) oligonucleotide possessing a total molecular weight (MW) of 23,299 Daltons. The concentration indicated that an optical density (OD) of 1.0 corresponded to 1.4 nanomoles. The libP1-76nt’s construction included a constant segment of 20 nt at either terminus, surrounding a central region of 36 randomized nucleotides. The terminal sequences had partial complementarity. Throughout the systematic evolution of ligands by exponential enrichment (SELEX), magnification of oligonucleotides was facilitated by two specific PCR primers, indicated as “S1” and “A2”. These primers were critical for the enrichment of both ssDNA and double-stranded DNA (dsDNA). Primer S1 was designed to extend and synthesize the complementary strand, containing a fluorescein amidite (FAM) label incorporated throughout PCR for ease of detection. In contrast, primer A2 was designed to create the antisense strand. The sequence of the random library was defined as 5′-TTCAGCACTCCACGCATAGC-N(36)-CCTATGCGTGCTACCGTGAA-3′, where “N” represented an equimolar mixture of the four deoxynucleotides adenine (A), guanine (G), thymine (T), and cytosine (C). Detailed sequences of the library and the associated primers are outlined in Supplementary Table S1.

### 4.3. MB-SELEX Procedure

#### 4.3.1. Preparation of MB-BSA and MB-HBP

Carboxylated magnetic beads (50 µL) were subjected to an activation process utilizing an activation solution. This activation solution was composed of a mixture of 50 µL of 0.4 mol/L EDC and 50 µL of 0.1 mol/L NHS, and the reaction was allowed to progress for 20 min at room temperature. Following activation, equimolar amounts of BSA and HBP at concentrations of 5 mg/mL and 300 µg/mL, respectively, were introduced to the activated beads. This mixture was incubated for 60 min at room temperature to allow for covalent coupling of the BSA (negative-screening protein) and HBP (positive-screening protein) to the beads.

After this incubation, beads were isolated from the unbound proteins with the use of a magnetic field, and the supernatant was decanted. The beads were blocked using 100 µL of ethanolamine (pH 8.5) for 10 min at room temperature to occupy any remaining active sites. Following blocking, the beads were rinsed four times using 200 µL of DPBS. During each wash, the beads were resuspended, mixed, and then pulled to one side of the container with a magnet to enable supernatant removal. The final preparations, including the magnetic beads conjugated with the BSA and HBP, were defined as MB-BSA and MB-HBP, respectively.

#### 4.3.2. MB-SELEX

In pursuit of tight binding and precise aptamers for human HBP, BSA was used as a counter-selection agent for negative screening to remove BSA-bound ssDNA from the library. Outlined in Supplementary Table S2, the aptamer selection process included 11 rounds of SELEX, commencing with positive screening, followed by 10 alternating rounds of positive and negative selection. In the initial selection, the single-stranded DNA library (Lib-76, 1.4 nmol) was constructed within 140 µL of PBS buffer, denatured at 95 °C for 5 min, and chilled on ice for 10 min to allow the proper conformation for binding. The ssDNA library was incubated with MB-BSA on a shaker for 60 min to eliminate sequences binding with BSA. Following this step, the library was introduced to MB-HBP for a 60 min incubation at room temperature. After binding, the MB-HBP complexes were rinsed four times using 200 µL of DPBS to exclude unbound oligonucleotides. The bound sequences were removed by heating along with 200 µL of DPBS, followed by centrifugation. The supernatant was obtained and defined as “R-Elution+”.

For amplification of the eluted sequences identified as R-Elution+, an ePCR system was constructed by adding 2 mL of PCR mix and 8 mL of microtiter-generating oil. The mixture was then subjected to ePCR. The resultant ePCR products were moved to 15 mL centrifuge tubes and concentrated using n-butanol. The cycling conditions were set as follows: initial denaturation at 95 °C for 3 min, followed by 30 cycles of denaturation at 95 °C for 1 min, annealing at 60 °C for 1 min, and extension at 72 °C for 1 min, using a final extension at 72 °C for 5 min, followed by an indefinite hold at 4 °C.

The dsDNA products were used to obtain single strands via denaturing urea PAGE. An 8% denaturing urea gel was generated using 1× TBE buffer, and the samples were loaded onto the gel. Electrophoresis was performed at 300 volts for 40 min. After the electrophoresis reaction, the gel was cut for the band of interest. The ssDNA was dialyzed overnight at 4 °C in PBS buffer. Quantification of the nucleic acid concentration was constructed using a NanoDrop-2000c spectrophotometer. The ssDNA was then used as a secondary library for the next round of screening, with each round of library denoted by R1 to R11.

To increase the selectivity of the aptamers, an initial positive screening was conducted, followed by alternating negative and positive screenings from the second round onwards. As outlined in Table S2 of the Electronic Supplementary Information (ESI), the selection pressure was increased by limiting the incubation time with the target cells from 60 min to 45 min and by strengthening the washing from four to six cycles.

The progression of the selection process was assessed using flow cytometry and qPCR. Upon verification of ssDNA pool enrichment, PCR was conducted using non-modified primers. The amplified ssDNA pool was sent to Aptamy Inc. (Hefei, Anhui, China) for high-throughput sequencing.

#### 4.3.3. Flow Cytometry Analysis

To assess the binding efficiency of the selected ssDNA pools and isolated aptamers, samples from R5, R8, and R11, as well as the aptamers, were diluted to a concentration of 500 nmol/L with PBS buffer. As controls, carboxylated MBs were developed in two sets: one activated and directly sealed (unmodified magnetic beads) and the other coupled with HBP (MB-HBP). The diluted ssDNA samples were denatured at 95 °C for 5 min, added to both the MB and MB-HBP, and incubated at room temperature for 60 min with shaking. After incubation, the beads were washed twice with DPBS buffer and resuspended in PBS buffer for analysis.

#### 4.3.4. Quantitative PCR for Library Enrichment

The initial Lib76 library was separated into templates using different dilutions (0, 10-4, 10-3, 10-2, 10-1) for qPCR and standard curve generation. To assess the enrichment of the libraries, qPCR was performed on the libraries from each round of screening to compute recovery rates. For qPCR analysis, 2 µL of the positively screened R-Elution+ was used in the reaction system. The qPCR cycling conditions were set as follows: an initial denaturation at 95 °C for 2 min, followed by 25 cycles of denaturation at 95 °C for 30 s, annealing at 60 °C for 30 s, and extension at 72 °C for 30 s. Subsequently, the length of the enriched libraries was verified using 8% PAGE.

#### 4.3.5. High-Throughput Sequencing and Sequence Analysis

R2, R4, R6, R8, R10, and R11 were diluted to a final concentration of 500 nmol/L and sent to Aptamy Inc. (China) for high-throughput sequencing and the degree of enrichment of the library was analyzed. The homology of the top 30 sequences was assessed utilizing ClustalX 2.1 and MEGA 7 software. Preservation and adaptation at specific sites were further assessed using the MEME suite. A phylogeny was developed to differentiate these sequences into families. From each family, the initial two sequences, each 76 nucleotides long, were selected for investigation. The affinity of each sequence to HBP was assessed using surface plasmon resonance (SPR) technology. Additionally, the Gibbs free energy of these aptamers was prognosticated using M-fold. These steps allowed for the selection of high-affinity nucleic acid aptamers for equilibrium dissociation constant (K_D_) determination.

#### 4.3.6. Affinity Assays by SPR

The binding affinity of the ssDNA pool and unique aptamers to HBP was quantified using a Biacore T200 SPR instrument (GE Healthcare, Chicago, IL, USA) at 25 °C. The aptamers were prepared in dilutions (1, 0.5, 0.25, 0.125, 0.0625, 0 µmol/L) using PBS buffer. CM5 sensor chips were activated through the injection of an equimolar mixture of EDC (0.4 mol/L) and NHS (0.1 mol/L) into channels 1 and 2. HBP (4 µg/mL) was introduced to channel 2 for immobilization, achieving a coupling level of 815 response units (RUs). After coupling, ethanolamine was utilized to block channels 1 and 2. For binding analysis, the diluted aptamers were placed into both channels at a flow rate of 30 µL/min for 150 s, followed by dissociation at the same flow rate for 600 s. Regeneration of the sensor surface was conducted using 4 mol/L NaCl at 30 µL/min for 130 s. Channel 1 was the reference flow cell, and the negative control was removed to establish the baseline. Data analysis was performed using BIA evaluation software version 3.0, with K_D_ determined using Langmuir 1:1 fitting models and BIA evaluation 4.0 software. Each experimental condition was performed in triplicate to ensure reproducibility.

#### 4.3.7. Specificity of the Aptamers

To examine the specificity of the selected aptamer candidates for HBP, binding assays were performed using a panel of inflammation-related proteins, including PCT, CRP, SAA1, and IL-6. Identical aptamer (500 nmol/L) and protein concentrations (300 µg/mL) were employed for flow cytometry following a previously described protocol.

#### 4.3.8. Circular Dichroism Spectrometer

The JASCO Circular Dichroism Spectrometer (model: JASCO, J-1700) was used to set the detection parameters and investigate the structural changes in the three aptamers (Apt-01, Apt-02, and Apt-13) upon interaction with the target protein HBP. The detection was performed by combining 100 μL of 5 μM aptamer solution with 100 μL of 5 μM HBP protein solution. Incubation at room temperature for 30 min preceded the analysis. To establish negative controls, we prepared two separate solutions: one containing 100 μL of 5 μM aptamer mixed with 100 μL PBS buffer (NaCl 137 mM, KCl 2.67 mM, Na_2_HPO_4_ 10 mM, KH_2_PO_4_ 2 mM, pH 7.4) and another consisting of 100 μL of buffer combined with a similar volume (100 μL) of a 5 μM protein solution.

### 4.4. Principle and Feasibility of Fluorescence Biosensor for HBP Based on the Selected Aptamer

In constructing a system to allow for the target-induced release of cDNA via aptamer recognition, designing an oligonucleotide to hybridize complementarily with a segment of the aptamer sequence was imperative. To assess the formation of dsDNA, this oligonucleotide was modified using FAM at the 3′ terminus and engineered to be partially complementary to the black hole quencher 1 (BHQ1)-labeled Apt-01. As outlined in Supplementary Table S3, five cDNAs (10 nt in length) were constructed to hybridize with Apt-01, identified as C1, C2, C3, C4, and C5, using the underlined segments as complementary sites. After the identification of the optimal complementary region for Apt-01, the length of this region was extended to 13 nucleotides to improve the hybridization stability. In addition, two adenines at the 3′ end of Apt-01 were removed, as described in Table S1. Subsequent to selecting C2-13, we determined the optimal concentration ratio between C2-13 and Apt-01(74) by detecting the fluorescence intensity of different ratios of C2-13 to Apt-01(74) (4:1, 2:1, 1:1, 1:2, 1:4). For hybridization reactions, equal volumes of BHQ1-labeled Apt-01(74) (200 nmol/L) and FAM-labeled C2-13 (200 nmol/L) were denatured, combined in the reaction system, and allowed to hybridize at 37 °C for 2 h. The preparation of the dsDNA was then assessed using a Hitche F-4600 fluorescence spectrometer (Hitachi Ltd., Tokyo, Japan) at an excitation wavelength of 485 nm and emission range of 500–600 nm. Both the excitation and emission slit widths were 5.0 nm. The proposed strategy was assessed using the fluorescence intensity at 520 nm. Experimental data were acquired at room temperature unless otherwise stated. Data were collected in triplicate, and the average value was utilized for quantitative analysis.

### 4.5. Characterization of Polyacrylamide Gel Electrophoresis

To examine the target-induced release mechanism of C2-13 from the dsDNA probe, the HBP was added to the 100 nmol/L Apt-01(74)/C2-13 probe. The aptamer, Apt-01(74), was binding to HBP and facilitating the freeing of C2-13. Exo III was added at 0.4 U to the reaction mixture, and the reaction was incubated at 37 °C for 2 h. Subsequent inactivation of Exo III was accomplished by heating to 80 °C for 10 min. After the reaction, the presence of ssDNA indicated successful C2-13 release. This was confirmed using 12% PAGE performed at 90 volts for 120 min.

To generate the circular template (CT), the padlock primer (PP) (2 µmol/L) and a 5′-phosphorylated padlock (2 µmol/L) were initially heat-treated at 95 °C for 5 min and allowed to cool to 37 °C over an hour. T4 DNA ligase (10 U) and the corresponding 1× T4 DNA ligase buffer were included in this mixture, which was incubated at 25 °C overnight to facilitate ligation. Following ligation, Exo III (10 U) and Exo I (5 U) were introduced to digest any remaining ssDNA and dsDNA. The digestion process was performed at 37 °C for 2 h. Enzymatic activity was stopped by heating the reaction to 80 °C for 10 min. The resultant circular DNA product was called a circular template (CT). The successful development of the CT was determined through 12% PAGE at 90 volts for 120 min post-preparation. The gel after electrophoresis was transferred to the imaging plate of a gel imager (Bio-Rad Ltd. Hercules, CA, USA) and the gel was imaged under UV light (the maximum absorption wavelength of SYBR Green I is about 497 nm and the maximum emission wavelength is about 520 nm).

12% PAGE (1 mm: 10× TBE, 600 μL. TEMED, 10 μL. 30% PAGE, 2.4 mL. H_2_O, 3.0 mL). Sample buffer: 6× loading buffer, 1 μL. 10× SYBR Green I, 1 μL. Run buffer: 1× TBE.

### 4.6. RCA-Based HBP Fluorescent Sensor

To develop the Apt-01(74)/C2-13 probe, Apt-01(74) (100 nmol/L) and C2-13 (100 nmol/L) were denatured by heating to 95 °C for 5 min, followed by a gradual cooling to 25 °C. The strands were then mixed and allowed to hybridize for 2 h at 37 °C. After hybridizing, various concentrations of HBP were introduced to the Apt-01(74)/C2-13 probe and incubated for 1 h to allow for Apt-01(74) binding and release of the C2-13 strand with an open 3′ end. Exo III at 0.4 U was then employed to digest any remaining dsDNA using a 2 h incubation at 37 °C. Enzymes were inactivated through heating the mixture to 80 °C for 10 min, obtaining the C2-13 strand. Following this, the CT (7.5 nmol/L) was incorporated into the system along with Phi29 DP (3 U), the corresponding 1× Phi29 DP buffer, and dNTPs (2 mmol/L). The rolling circle amplification (RCA) reaction was performed by incubation at 37 °C for 15 min. For fluorescence characterization, 4 µL of 20× SYBR Green II was included within the reaction, which was then assessed at an excitation wavelength of 495 nm and emission range of 505–600 nm after 15 min at room temperature in the darkness.

### 4.7. Selectivity of the Sensor

The specificity of the assay was identified through a comparison of the fluorescent responses elicited by the HBP against other inflammatory markers, such as PCT, CRP, IL-6, and SAA1, as well as a composite mixture of these proteins. The inflammatory protein was introduced to the Apt-01(74)/C2-13 at 5 nmol/L, followed by a 60 min incubation at room temperature. The protocol described earlier was utilized, involving Exo III addition, RCA, and SYBR Green II incorporation. The alterations in fluorescence intensity were recorded to assess the assay’s selectivity.

### 4.8. Analytical Application in Real Samples

In order to verify the accuracy and the practical effectiveness of the fluorescence sensor constructed in this study, with the consent of patients, blood samples were collected from 2 healthy individuals undergoing physical examinations and 5 sepsis patients from the Fuzhou General Teaching Hospital (the 900th Hospital). After centrifugation, the fluorescence sensor was used to detect HBP in serum (initial approval date: 19 July 2023, IRB number 2023-061).

## 5. Conclusions

Three ssDNA aptamers that specifically bind HBP using the MB-SELEX strategy were screened. These aptamers had a high affinity for HBP, with K_D_ values of 3.42 nmol/L, 1.44 nmol/L, and 1.04 nmol/L, respectively. A novel fluorescent biosensor for HBP was developed by the screened aptamer, which exhibits a linear detection range from 0.01 nmol/L to 10 nmol/L and a detection limit of 0.0056 nmol/L. Furthermore, the biosensor showcased remarkable specificity by not interacting with four other common inflammation-associated proteins. This study validates the feasibility and reliability of the HBP-targeted aptamer for the accurate detection of HBP in complex sample matrices and offers a foundational framework for the development of nucleic acid aptamer-based sepsis detection.

## Figures and Tables

**Table 1:** Analysis result of the biosensor on sepsis patients.

Sample	Detected HBP (nmol/L)	Clinical Diagnosis
blank	0	/
1	0.24	Healthy
2	0.22	Healthy
3	2.68	Sepsis
4	6.1	Sepsis
5	2.88	Sepsis
6	6.32	Sepsis
7	3.57	Sepsis

Note: The samples include PBS buffer (Blank), serum from healthy (1 and 2), and serum from sepsis patients (3 to 7).

## Data Availability

The data supporting the findings of this study are available in the Supplementary Material of this article.

## Supplementary Materials

The following supporting information can be downloaded at: https://www.mdpi.com/article/10.3390/molecules29081717/s1. Figure S1: Characterization of the library. Standard curves for initial libraries alongside log values of their concentrations. Figure S2: Following sequencing, the initial 30 sequences were examined for homology and separated into seven families according to their homolog. Figure S3: Conserved and variant site assessment of the top 30 sequences with comparatively high abundance. Figure S4: Modeling of the secondary structure of three aptamers using Mfold. Figure S5: Aptamer and HBP of CD spectra in PBS buffer. Figure S6: The FRET effect occurred following the hybridization of FAM modified C2-13 with BHQ1 modified Apt-01. Figure S7: Characterization of CT formation by polypropylene gel electrophoresis. Table S1: Libraries, primers and probe sequences. Table S2: Control the incubation time and number of wash time of the screen to ensure the affinity of the library to the target. Table S3: Five kinds of cDNA were designed to hybridize to each of the five regions of Apt-01 to explore the regions that bind to aptamer. Sequences underlined are the Apt-01 complementary sites to the cDNA. Table S4: High-throughput sequencing top 100 sequence results. Table S5: Stability and affinity characterization of 14 candidate aptamers.
