# Supplementary material for: Screening and Application of DNA Aptamers for Heparin-Binding Protein
Source: Molecules. 2024 Apr 10;29(8):1717. doi: 10.3390/molecules29081717 (PMC11051826; doi:10.3390/molecules29081717)
Supplement: Supplementary file 1 [file molecules-29-01717-s001.zip › molecules-2929405-supplementary.pdf]

Supporting Information

Screening and Application of DNA Aptamers for Heparin-Binding Protein

Xi Zhou, Yingying Cao, Xiaocui Huang, Shuqian Qiu, Xinran Xiang, Huimin Niu, Li Chen, Shuiliang Wang, Zhenyu Li and Shenghang Zhang

SECTION A: SUPPORTING TABLES

Table S1. Libraries, primers and probe sequences.

Name	Sequence （5'-3'）
Upstream fixed sequence	TTCAGCACTCCACGCATAGC
Downstream fixed sequence	CCTATGCGTGCTACCGTGAA
S1	FAM - TTCAGCACTCCACGCATAGC
A2	PloyA (19) - Spacer 18 - TTCACGGTAGCACGCATAGG
Apt-01(74)	TTCAGCACTCCACGCATAGCTTCACCTACCGCAATCCGTTGCTCA GTGCTACCGCACCTATGCGTGCTACCGTG
C2-13	CTTCACGGTAGCACGC
Apt-01(74)-BHQ1	TTCAGCACTCCACGCATAGCTTCACCTACCGCAATCCGTTGCTCA GTGCTACCGCACCTA/iBHQ1dT/GCGTGCTACCGTG
C2-13-FAM	CTTCACGGTAGCACGC - FAM
Padlock primer	GACTGGGCCTCAGTCCGTTTCTCATGGTTC
Padlock	5'P- GACTGAGGCCCAGTCCTTGAATTTGACGCGTGCTACCGTGAAGG AACCATGAGAAACG

Note: Sequences of letters with corresponding underscores or italics are complementary.

Table S2. Control the incubation time and number of wash time of the screen to ensure the affinity of the library to the target.

SELEX rounds	Negative SELEX		Positive SELEX	
	Incubation time/min	wash times	Incubation time/min	wash times
1			60	4
2	60	4	60	4
3	60	4	60	4
4	60	4	60	4
5	60	4	60	4
6	60	4	60	4
7	60	4	60	4
8	60	4	60	4
9	60	4	45	6
10	60	4	45	6
11	60	4	45	6

Table S3. Five kinds of cDNA were designed to hybridize to each of the five regions of Apt-01 to explore the regions that bind to aptamer. Sequences underlined are the Apt-01 complementary sites to the cDNA.

cDNA name	Sequence (5'-3')	Aptamer (5'-3')
C1	TTCACGGTAG-FAM	TTCAGCACTCCACGCATAGCTTCACCTACCGCAATCCGTTGCTCAGTGCTACCGCACC/iBHQ1dT/ATGCGTGCTACCGTGAA
C2	GGTAGCACGC-FAM	TTCAGCACTCCACGCATAGCTTCACCTACCGCAATCCGTTGCTCAGTGCTACCGCACC/iBHQ1dT/ATGCGTGCTACCGTGAA
C3	CATAGGTGCG-FAM	TTCAGCACTCCACGCATAGCTTCACCTACCGCAATCCGTTGCTCAGTGCTACCGCACC/iBHQ1dT/ATGCGTGCTACCGTGAA
C4	ATTGCGGTAG-FAM	TTCAGCACTCCACGCATAGCT/iBHQ1dT/CACCTACCGCAATCCGTTGCTCAGTGCTACCGCACCTATGCGTGCTACCGTGAA
C5	TGAAGCTATG-FAM	TTCAGCACTCCACGCATAGCT/iBHQ1dT/CACCTACCGCAATCCGTTGCTCAGTGCTACCGCACCTATGCGTGCTACCGTGAA

Note: Sequences of letters with corresponding underscores complement each other.

Table S4 High-throughput sequencing top 100 sequence results.

Number	Random sequence (5'-3')	R11
1	TTCACCTACCGCAATCCGTTGCTCAGTGCTACCGCA	11404
2	TTCACCTACCGCGCAGCTCAGTCGTTGCCCACACGA	11608
3	TTCACCTACGAACGCCAGTGACAGCACCGATCAGTG	9823
4	TTCATCTACCCCGCCAAGGTCAGCCACGTCAGTGTG	8127
5	TTCACCTACACCGCGACCGCGTTCAGCCTCTGCAGG	8966
6	TTCATCTACCGCGTTAGCTCAGTCGTCTCGTAACGA	8945
7	CTTGCCTACCGGACGAAGTACTCAGTCACTCCGTGA	5902
8	TCATCGCGAGCGACACGTTACCGTGCTTTACTACCG	8462
9	CTTGCCTACCGATCCTGGATCTCAGCTGTACCACAG	7003
10	CTTGCCTACCCACAGCGTGTCAGTGTACTGCGTACA	2379
11	TTTACTACCGCGCACTTAGTCGCTCAGCCGTTCCGG	5812
12	TTCACCTACTGGCATATGCCTCAGTCCACTCCTGGA	5850
13	TCATCGCCTGCGACATGCTAGCATGCTTTACTACTG	5695
14	TTCACCTACCGCCAATCAGCTCAGTGTGTCTCCACA	3640
15	TTCACCTACTGCCCTAGCTCAGCCGTCTCCATCCGG	5248
16	TTTACCTACCGCGCAGCTCAGTCGTTGCCCACACGA	5810
17	CTTGCCTACCGACAACCGTCTCAGTTCGTCATCGAA	4828
18	TTTACCTACCGCATCGCTCAGTCAACCATTGGTGGA	4631
19	TCATGCCCTGTGCACGGCCCACCCGCTTTACCTACG	3666
20	TTCACCTACCGCCGAAGGCTCAGTCCGTGCTTCGGA	6199
21	TTTACCTACTGCCACAACTGGGGCTCAGCCGCGAGG	5609

22	TCATCCGGATCGGACACATCTTGTGCCTTGCTACTG	2286
23	TTCACCTACCGCCCTGCTCAGCCGACTCGGATCGG	5004
24	TTCATCTACCGCGAGCAGCTCAGCATGCCCAGCATG	3454
25	TTCACCTACTGCCACAACTGGGGCTCAGCCGCGAGG	2844
26	TTCATCTACCGTGCCTCTCGCACTCAGCCCCTGTGG	4138
27	TCATCCCATGGGACCGTTATACGGCTTCACCTACCG	2718
28	TTCATCTACTGCGACTAGCTCAGCCTATCCACTAGG	3825
29	TTCATCTACCGCACCAGCTCAGCCGACCTCCGGAGG	3514
30	CTTGCTACCGGGAACCTCAGTCAGTGTGTCAACTGA	2449
31	TTCACCTACTGCCTCGGCTCAGATTCGATTCCGAAT	2987
32	TTCACCTACCGCCTTGCTCAGCCCATTCGGAATGGG	2217
33	TTCACTACCGCGCTAAGCTCAGTTTGGGTGACCAAA	1733
34	CTTGTCTACCCGGGAACCGTCAGTCCGTCACACGGA	1824
35	TTTACCTACCGCAATCCGTTGCTCAGTGCTACCGCA	2915
36	TTCATCTACCGCCCCAGCTCAGCCGACCTCCGGAGG	3092
37	CTTGCTACCGTCCTTGACTCAGTTCAGCGCCGTGAA	1275
38	CTTGCCTACCGGGAGAATCCCTCAGACACAGCGTGT	1278
39	TTTACTACGACCACCGGGACAGATTGTGCAGACGAT	1667
40	TTCATCTACCGGTCGCGAGACCTCAGTCACACGTGA	1914
41	CTTGCTACGAGCCTGCGACAGTCGCAAACTTTGCGA	1741
42	TTTACTACCGCCCTGGCTCAGATGGTTCCCGGCCAT	1319
43	CTTGCCTACCCACACCGTGTCAGTCTGTACGTCAGA	2596
44	TTCACCTACGAGCGCAGTGACAGTGGTCGCAGTCCA	1449
45	TTTACCTACCGCCCTGCTCAGCCGACTCGGATCGG	2896
46	TTTACCTACCGCACCTCGGGCTCAGTTGCATGACAA	2289
47	TTTACCTACCGCACCCGCTCAGTCGTCCTCCGCGA	2777
48	TTCACCTACCGCACCTCGGGCTCAGTTGCATGACAA	1007
49	TTCACCTACCGCTACCCTGCTCAGCTGCCAAAGCAG	2220
50	CTTGCCTACCGACATTGTTGTCTCAGTTCCTACGAA	1579
51	CTTGCCTACTGCCTATCGGGCTCAGTCGTTCCTCGA	1850
52	TTCACCTACCGCTGTGATTAGCAGCTCAGCCATCGG	2664
53	TTCATCTACCGCGCAGCTCAGTCGTTGCCCACACGA	2252
54	TTCACCTACCGCGCAGATCGCTCAGCCACTCATTGG	1930
55	TTTACTACCGCCACATGCTGGCTCAGTCCCTGAGGA	1624
56	CTCGCCTACCGACAACCGTCTCAGTTCGTCATCGAA	811
57	TTCACTACCGCGCCAGCTCAGTCGGACCAGCTGCGA	944
58	TTTACCTACCGCCGTGCTCAGTTGGACTTGTCCCAA	1772
59	CTTGCCTACCGCGCCAGCTCAGGGAACGTACCGTTGC	1536
60	CTTGCTACACCGACAACGTCGTTCAGTGGTCCACCA	955
61	CTTGTCTACCCGCCTACTGGACGTCAGCTCGTTGAG	837
62	TCATTGCCCCGCAACAACTCGTTTGCTTCATCTACG	1240
63	TTTACCTACTGCGGAACGCTCAGACGATGCCACCGT	1270
64	TTTACCTACCTCGCAGTCCGCCCGCTTGGCATCGGA	861
65	CTTGCTACCGTCGCCGACTCAGTTGGATTTGCCCAA	894

66	CTTGCTACCTCGACAGATCAGTCACACGCCCTGGGA	641
67	CTTGCCTACCGCATCACATGCTCAGTGTCCGCGACA	2066
68	TTTACCTACCGCGATCGCTCAGTCCGCCTTGCTGA	2315
69	CTTGCCTACTGGCCCCGCTGCCTCAGTGTTCTAACA	1038
70	CTTGCCTACCGTCCTGAACTCAGTCTCGTTCCGAGA	851
71	TTTACCTACCGCCGTGGCTCAGTTGTCTAGGAACAA	1333
72	CTTGCTACTCGGAACGTCAGCTCGCACTCCCGCGAG	560
73	TCATCGTCCCGGACGACGTCCCGCCTTGTCTACCG	932
74	TTTACCTACCGCGATCAGCTCAGTCTCACCGTGAGA	2181
75	CTTGCTACGAGTCTGAACGACAGTCCGGTTGACGGA	630
76	TTTACCTACCGCCAATCAGCTCAGTGTGTCTCCACA	1545
77	TCATCCGCTCGGACGGTGACCACCGCTTTACCTACG	2025
78	TTTACCTACTGCCCCCGCTCAGTCACGCCACCGTGA	2018
79	CTTGCTACCCGCGAATAGCGTCAGTCGGCCTTGCGA	341
80	CTTCCTGGTGTCGGGCAGTTATAACGGTGCGTCTGA	423
81	CTTGCCTACCCCGGTCTCGGTCAGTCGTTGTGTCGA	330
82	CTTGCCTACCGATCTAGGATCTCAGTGTTGCCGACA	783
83	TTTACCTACCGCGAGGTACCGACGCTCAGCCTTTGG	1743
84	TTTACCTACCGCGGATTCCTGCTCAGTCTCCAGAGA	1632
85	TTCACCTACTGCCTTAGCTCAGGTCTGCATACTGAC	889
86	TTTACCTACCGCACCGACGTCGCTCAGCCCTTGGG	1785
87	TTCATCTACCGCATCCCGCTCAGTCGGCCGGACCGA	1483
88	TCATCGGCACGACCGCTATTGCTGCTTTACCTACCG	1479
89	CTTGCTACCGTTCCTACCCTGAACTCAGTCCGTGGA	455
90	CTTGCCTACCGCGACTCGATCTCGCTCAGTCTCTGA	911
91	CACTTCACCTACGACCACCGGGACATCGCGCTCGATG	482
92	TTCACCTACTGCCTAGCTCAGCCGTCTCCATCCGG	1590
93	TTCATCTACCGGTCCCACAGACCTCAGTGCGTCGCA	1278
94	TTTACCTACCGCACACTGTTGCTCAGTCGCTTTCGA	1680
95	TTCACCTACCGCGCCAGCTCAGGGAACGTACCGTTGC	1355
96	TTTACTACCGTGGCCACACTCAGTTGCAGTTCGCAA	942
97	TTCATCTACGACCTCCCGGGACAGTCACGCCTGTGA	1049
98	TTCACCTACCGCGTTAGCTCAGTCGTCTCGTAACGA	1315
99	TTTACCTACCTCGCAGTCTGTCAGCACGTTGCCAGA	737
100	CTTGCTACCGAGCCGTGCTCTCAGTTCCTTCCAGAA	687

Note: R11 denotes the number of corresponding sequence entries in the eleventh round of screening libraries.

Table S5 Stability and affinity characterization of 14 candidate aptamers.

Aptamer	Full-length sequence (5'-3')	Family	ΔG (kcal/mol)	ΔH (kcal/mol)	ΔS (kcal/mol)	Tm (°C)	RU
Apt-01	TTCAGCACTCCACGCATAGCTTCACCTACCGCAATCCGTTGCTCAGTGCTACCGCACCTATGCGTGCTACCGTGAA	5	-12.48	-145.20	-445.15	53.0	424.1
Apt-02	TTCAGCACTCCACGCATAGCTTCACCTACCGCGCAGCTCAGTCGTTGCCCACACGACCTATGCGTGCTACCGTGAA	5	-11.43	-134.50	-412.78	52.7	161.7
Apt-03	TTCAGCACTCCACGCATAGCTTCACCTACGAACGCCAGTGACAGCACCGATCAGTGCCTATGCGTGCTACCGTGAA	3	-11.67	-131.10	-400.57	54.1	118.6
Apt-04	TTCAGCACTCCACGCATAGCTTCATCTACCCCGCCAAGGTCAGCCACGTCAGTGTGCCTATGCGTGCTACCGTGAA	3	-9.16	-139.80	-438.17	45.9	121.1
Apt-05	TTCAGCACTCCACGCATAGCTTCACCTACACCGCGACCGCGTTCAGCCTCTGCAGGCCTATGCGTGCTACCGTGAA	2	-12.07	-138.80	-425.05	53.4	132.2
Apt-07	TTCAGCACTCCACGCATAGCCTTGCCTACCGGACGAAGTACTCAGTCACTCCGTGACCTATGCGTGCTACCGTGAA	6	-10.24	-143.60	-447.29	47.9	76.2
Apt-08	TTCAGCACTCCACGCATAGCTCATCGCGAGCGACACGTTACCGTGCTTTACTACCGCCTATGCGTGCTACCGTGAA	1	-12.14	-129.80	-394.63	55.8	79
Apt-09	TTCAGCACTCCACGCATAGCCTTGCCTACCGATCCTGGATCTCAGCTGTACCACAGCCTATGCGTGCTACCGTGAA	6	-11.14	-141.60	-437.57	50.5	87.5

Apt-12	TTCAGCACTCCACGCAT AGCTTCACCTACTGGCA TATGCCTCAGTCCACTC CTGGACCTATGCGTGCT ACCGTGAA	7	-13.07	-144.00	-439.14	54.8	107.2
Apt-13	TTCAGCACTCCACGCAT AGCTCATCGCCTGCGAC ATGCTAGCATGCTTTACT ACTGCCTATGCGTGCTA CCGTGAA	1	-11.76	-129.10	-393.56	54.9	169.4
Apt-14	TTCAGCACTCCACGCAT AGCTTCACCTACCGCCA ATCAGCTCAGTGTGTCT CCACACCTATGCGTGCT ACCGTGAA	4	-11.34	-129.50	-396.31	53.6	50.3
Apt-15	TTCAGCACTCCACGCAT AGCTTCACCTACTGCCC TAGCTCAGCCGTCTCCA TCCGGCCTATGCGTGCT ACCGTGAA	2	-11.21	-125.00	-381.65	54.4	72.5
Apt-18	TTCAGCACTCCACGCAT AGCTTTACCTACCGCAT CGCTCAGTCAACCATTG GTGGACCTATGCGTGCT ACCGTGAA	7	-9.57	-103.30	-314.37	55.4	41.2
Apt-20	TTCAGCACTCCACGCAT AGCTTCACCTACCGCCG AAGGCTCAGTCCGTGCT TCGGACCTATGCGTGCT ACCGTGAA	4	-15.44	-146.80	-440.58	60.0	99

Note: The Gibbs free energy showed that the 14 selected candidate aptamers were all stable with little variation between sequences, but seven candidate aptamers showed response values (RU) of more than 100 RU to HBP, of which Apt-01, Apt-02, and Apt-13 showed response values of more than 150 RU.

SECTION B: SUPPORTING FIGURES

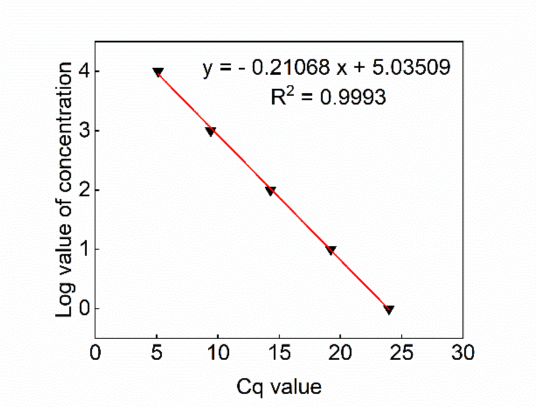

Figure S1. Characterization of the library. Standard curves for initial libraries alongside log values of their concentrations.

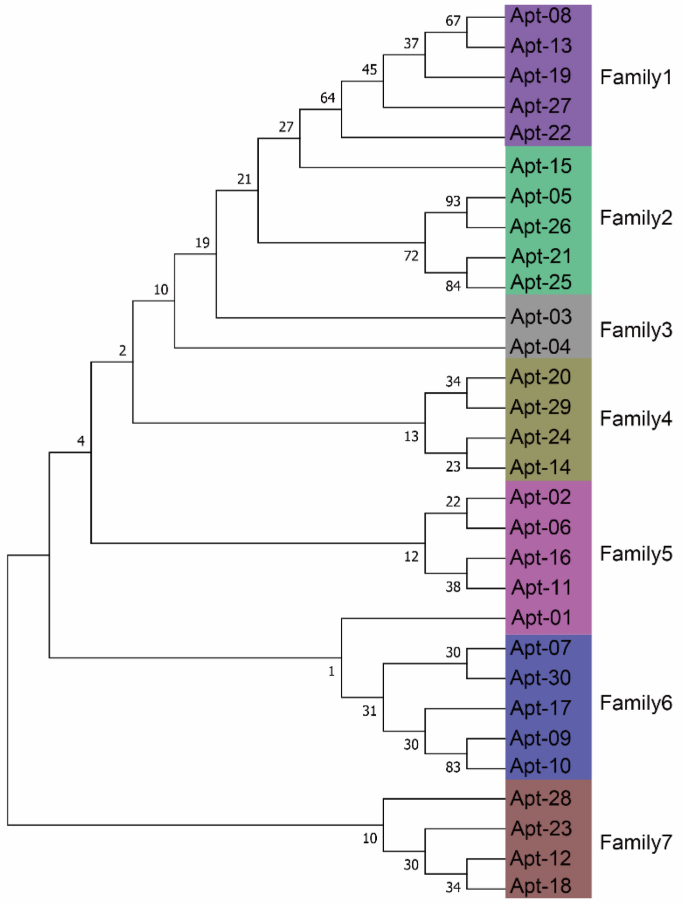

Figure S2. Following sequencing, the initial 30 sequences were examined for homology and separated into seven families according to their homolog.

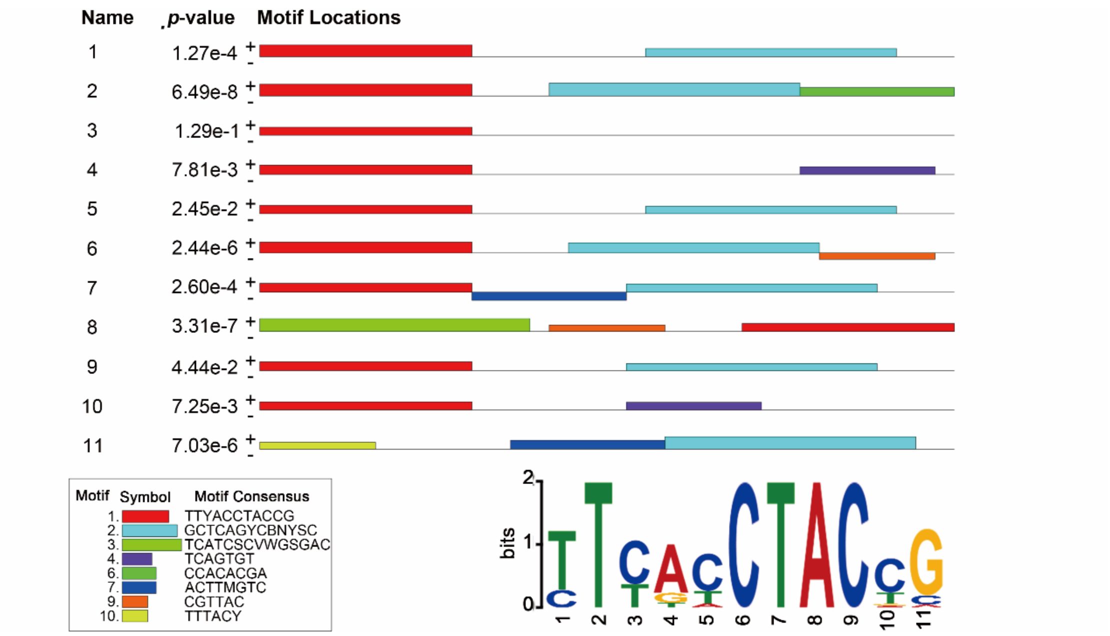

Figure S3. Conserved and variant site assessment of the top 30 sequences with comparatively high abundance.

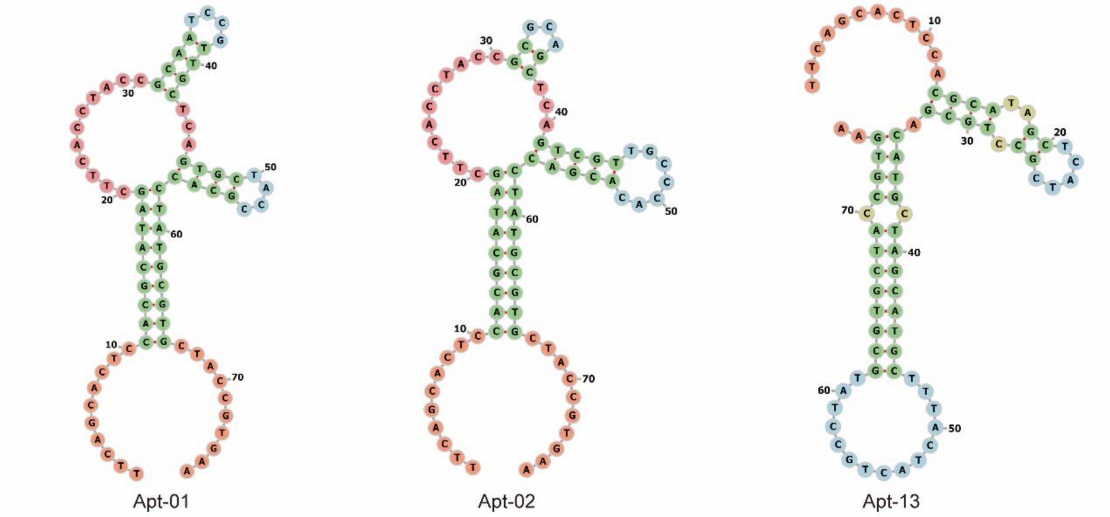

Figure S4. Modeling of the secondary structure of three aptamers using Mfold.

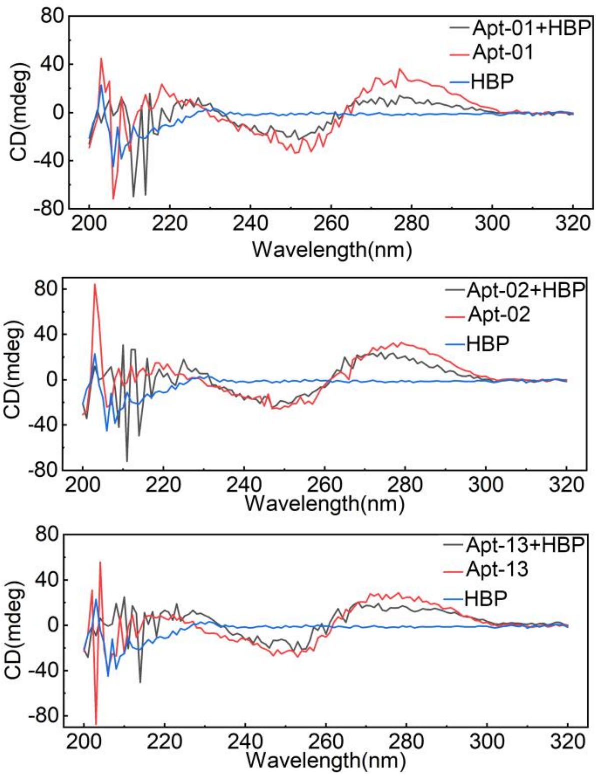

Figure S5. Aptamer and HBP of CD spectra in PBS buffer.

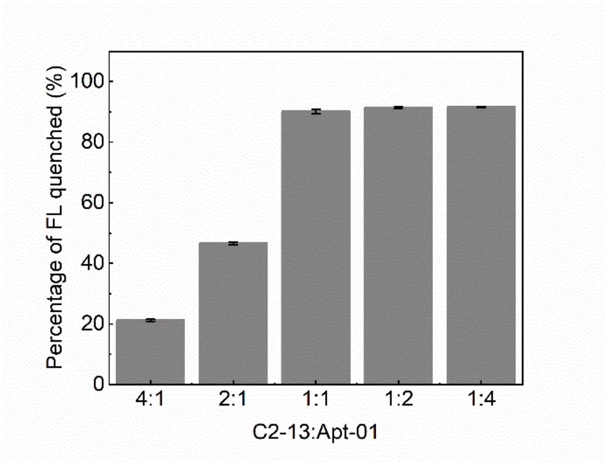

Figure S6. The FRET effect occurred following the hybridization of FAM modified C2-13 with BHQ1 modified Apt-01.

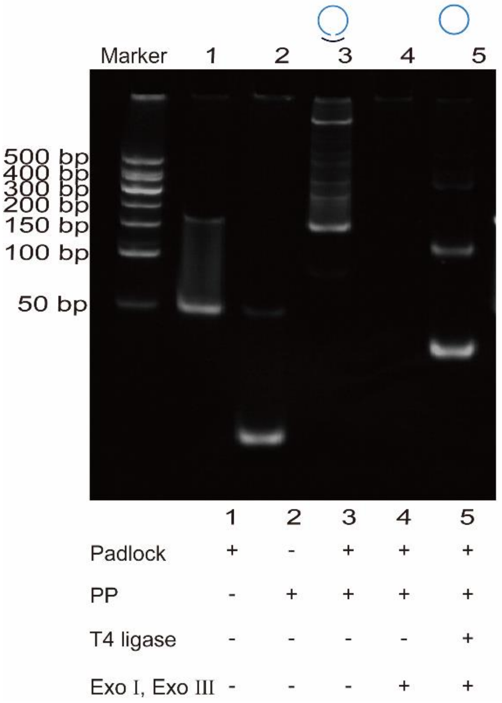

Figure S7. Characterization of CT formation by polypropylene gel electrophoresis.
